# Disease-Dependent Local IL-10 Production Ameliorates Collagen Induced Arthritis in Mice

**DOI:** 10.1371/journal.pone.0049731

**Published:** 2012-11-16

**Authors:** Louise Henningsson, Tove Eneljung, Pernilla Jirholt, Sara Tengvall, Ulf Lidberg, Wim B. van den Berg, Fons A. van de Loo, Inger Gjertsson

**Affiliations:** 1 Department of Rheumatology and Inflammation Research, Institute of Medicine, University of Gothenburg, Gothenburg, Sweden; 2 Sahlgrenska Center for Cardiovascular and Metabolic Research, Wallenberg Laboratory, Sahlgrenska University hospital, Gothenburg, Sweden; 3 Rheumatology Research and Advanced Therapeutics, Radboud University Nijmegen Medical Centre, Nijmegen, The Netherlands; University Hospital Jena, Germany

## Abstract

Rheumatoid arthritis (RA) is a chronic destructive autoimmune disease characterised by periods of flare and remission. Today’s treatment is based on continuous immunosuppression irrespective of the patient’s inflammatory status. When the disease is in remission the therapy is withdrawn but withdrawal attempts often results in inflammatory flares, and re-start of the therapy is commenced when the inflammation again is prominent which leads both to suffering and increased risk of tissue destruction. An attractive alternative treatment would provide a disease-regulated therapy that offers increased anti-inflammatory effect during flares and is inactive during periods of remission. To explore this concept we expressed the immunoregulatory cytokine interleukin (IL)-10 gene under the control of an inflammation dependent promoter in a mouse model of RA - collagen type II (CII) induced arthritis (CIA). Haematopoetic stem cells (HSCs) were transduced with lentiviral particles encoding the IL-10 gene (LNT-IL-10), or a green fluorescence protein (GFP) as control gene (LNT-GFP), driven by the inflammation-dependent IL-1/IL-6 promoter. Twelve weeks after transplantation of transduced HSCs into DBA/1 mice, CIA was induced. We found that LNT-IL-10 mice developed a reduced severity of arthritis compared to controls. The LNT-IL-10 mice exhibited both increased mRNA expression levels of IL-10 as well as increased amount of IL-10 produced by B cells and non-B APCs locally in the lymph nodes compared to controls. These findings were accompanied by increased mRNA expression of the IL-10 induced suppressor of cytokine signalling 1 (SOCS1) in lymph nodes and a decrease in the serum protein levels of IL-6. We also found a decrease in both frequency and number of B cells and serum levels of anti-CII antibodies. Thus, inflammation-dependent IL-10 therapy suppresses experimental autoimmune arthritis and is a promising candidate in the development of novel treatments for RA.

## Introduction

Rheumatoid arthritis (RA) is a systemic chronic autoimmune disease that mainly affects the joints and ultimately leads to severe bone and cartilage destruction. The clinical course of the disease is discontinuous and characterised by spontaneous remissions and exacerbations. The aetiology in RA is largely unknown but for some reason the immune system - which normally protects us against exogenous pathogens - is dysregulated and has lost its normal tolerance to endogenous (self-) structures and mediates an inflammatory attack against e.g. the joints. Todays treatment is based on continuous immunosuppression either by conventional disease modifying anti-rheumatic drugs such as methotrexate and/or by biological agents targeting specific proteins e.g. TNF. Unfortunately these treatment modalities can cause side effects such as severe infections and, in addition, attempts to withdraw therapies in established RA often leads to flares [1]. To overcome these hurdles, disease-regulated therapy appears ideally suited, as it would allow intrinsic expression of the immunosuppressive therapy only during inflammatory conditions i.e. during disease flares but not during periods of remissions. This approach has been used successfully in experimental autoimmune encephalomyelitis (EAE) where, by means of transcriptionally targeted gene therapy, a T cell targeted IL-2 promoter controlling IL-10 production delayed onset and progression of EAE [2]. It has also been shown that disease-regulated IL-4 expression achieved via the IL-1/IL-6 promoter can protect against cartilage destruction in CIA [3].

Interleukin-10 is produced by a multitude of cell types during an immune response, where one of its main functions is to limit the ongoing response in order to protect the host from excessive immune mediated tissue destruction (reviewed in [4]), which is one of the characteristics in RA. Support for a role of IL-10 in RA comes from mouse models: in the CIA model, treatment with anti-IL-10 antibodies aggravates the disease, as does a complete lack of IL-10 [5], [6]. This argues for IL-10 as a possible cytokine to use for treatment of RA. Indeed, addition of recombinant IL-10 [7], transfer of IL-10 producing cells [8] or continuous production of IL-10 [9], [10], [11], reduces the severity but not the frequency of CIA. However, a permanent increase in IL-10 levels may not be optimal as it may also influence defence towards invading pathogens whereas an increase exclusively during inflammation (flares) would be preferable and could provide a treatment alternative in CIA and RA.

Inflammation induced IL-10 transcription in endothelial cells, driven by an E selectin promoter, has been used by Garaulet et al. and showed promising results in ameliorating arthritis [12]. We sought to investigate whether IL-10 expression induced by a promoter sensitive to pro-inflammatory cytokines IL-6 and IL-1 in haematopoetic cells, could be a candidate for tailor-made therapy for CIA and with a long term goal also for RA patients. Our data show that inflammation-induced local expression of IL-10 delays progression of CIA through decreased serum levels of IL-6 and anti-CII antibodies. This study provides evidence that inflammation-dependent immunosuppression is a promising tool for the treatment of autoimmune arthritis.

## Results

### Arthritis is Ameliorated in LNT-IL-10 Mice

**Figure 1:**
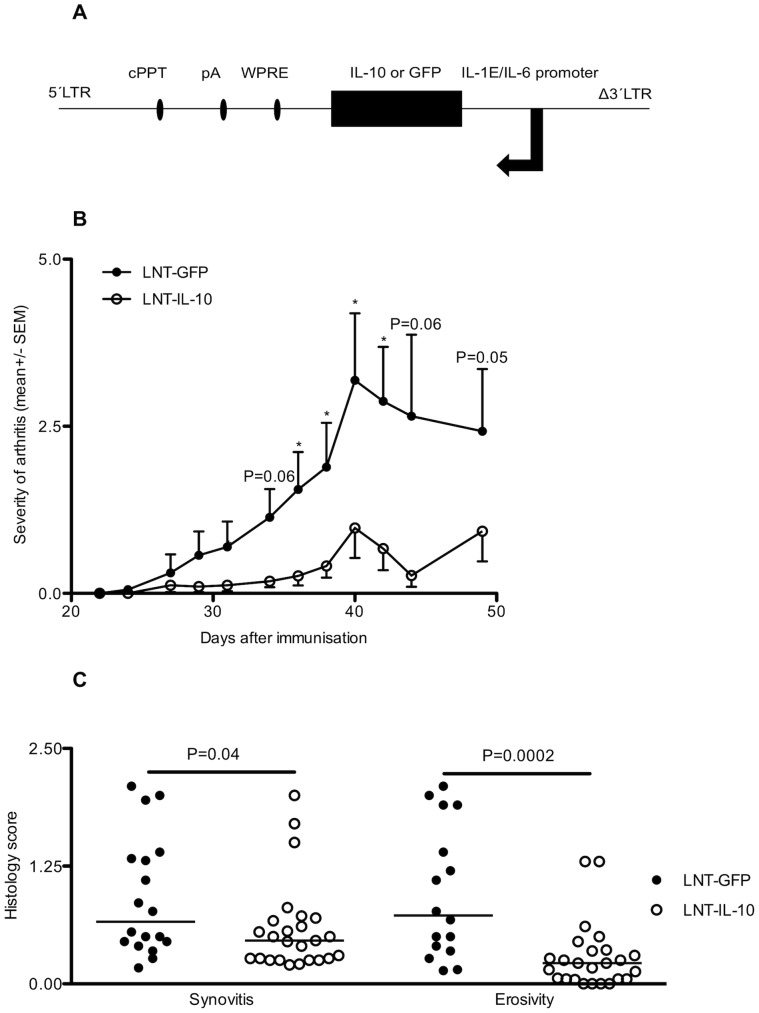
Lentiviral gene constructs and clinical development of arthritis. (**A**) Lentiviral constructs: LNT-GFP and LNT-IL-10. LTR; long terminal repeat, cPPT; central polypurine tract, pA; polyadenylic acid tail, WPRE; Woodchuck post-transcriptional regulatory element, IL-1E; Interleukin-1 enhancer, IL-6 promoter. (**B**) Severity of arthritis (mean arthritis score ± SEM). LNT-GFP (day 0–42 n = 18, day 44–49 n = 10) and LNT-IL-10 (day 0–42 n = 25, day 44–49 n = 14)). (**C**) Histopathological severity of synovitis and cartilage and bone erosivity measured as histological severity score (Y-axis) ranging from 0–3. Data in figure 1B and C were analysed by Mann-Whitney U-test. Closed circles represents LNT-GFP and open circles LNT-IL-10 mice. Bars in 1C represent the median.

To investigate whether an inflammation-dependent increase in IL-10 production would change disease status in the CIA mouse model, the promoter of the human cytokine gene interleukin-6 (*IL-6*) in combination with the IL-1 enhancer region [13] were used to drive the expression of the IL-10 (LNT-IL-10) or a green fluorescent (LNT-GFP) control gene (Figure 1A). We find that this vector gives rise to inflammation-dependent IL-10-production *in vitro* (Suppl 1A). Haematopoetic stem cells (HSCs) were transduced with LNT-IL-10 or LNT-GFP lentiviral particles and were thereafter injected into lethally irradiated recipient mice. After the HSCs had re-populated the immune system, CIA was induced (12 weeks post transplantation). The integration of the lentiviral construct was successful (Suppl 1B) and, although almost all mice in both groups developed arthritis, those transplanted with the LNT-IL-10 transduced HSCs showed a significant reduction in the severity of clinical arthritis (Figure 1B). Importantly, the histology showed reduced synovitis, and cartilage and bone erosivity in the LNT-IL-10 mice compared with controls (Figure 1C). Thus, integration of the LNT-IL-10 construct containing the IL-10 gene under the regulation of an inflammation-dependent promoter suppresses the progression of CIA.

### LNT-IL-10 Treatment Increases IL-10 and SOCS1 Expression in Lymph Nodes

**Figure 2:**
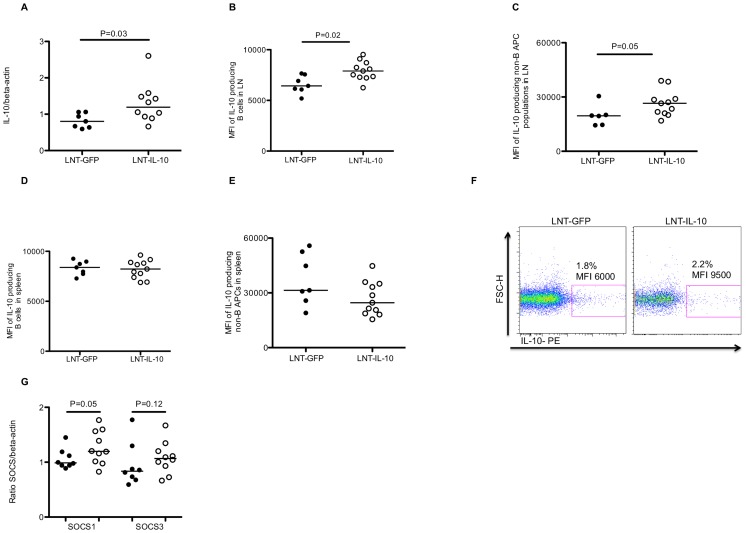
Levels of IL-10 mRNA, intracellular IL-10 production and SOCS expression (**A**)**.** Levels of IL-10 mRNA expression in lymph nodes at day 42 in LNT-GFP or LNT-IL-10 mice. (**B**) The amount of IL-10/cell measured as geometric mean flourescent intensity (MFI) in lymph node CD19^+^MHC II^+^B cells, (**C**) in lymph node CD19^-^MHC II^+^non-B APCs (**D**) in splenic B cells, (**E**) in splenic non-B APCs. (**F**) Typical gating for intracellular cytokine staining showing one sample from an LNT-GFP mouse and an LNT-IL-10 mouse (**G**) Levels of mRNA SOCS1 and 3 expression in draining lymph nodes at day 42. In figure 2A–E and G data were analysed by Mann-Whitney U-test. Closed circles represents LNT-GFP and open circles LNT-IL-10 mice.

The reduction in arthritis in LNT-IL-10 mice suggested that IL-10 is produced. To investigate this, mRNA expression levels of IL-10 were analysed in draining lymph nodes where, as expected, IL-10 mRNA levels were increased in LNT-IL-10 mice compared with controls (Figure 2A). In addition, flow cytometric analysis of lymph node cells from LNT-IL-10 mice at termination of the experiment, showed a significant increase in the amount of IL-10 per cell compared to control mice, measured as mean fluorescence intensity (MFI), though there was no difference in the number of IL-10-producing cells (data not shown). Gating on different cell populations demonstrated that IL-10 was in particular produced by B cells, and by non-B antigen presenting cells (APC) (Figure 2 B, C and 2F). The proportion and expression (MFI) of IL-10 in B cells and non-B cell APCs in spleen were similar between the groups (Figure 2 D–E). Analysing IL-10 in serum by ELISA showed similar levels in both groups of mice (data not shown). Taken together this suggests that IL-10 acts locally in the lymph nodes rather than on a systemic level.

To investigate the link between increased IL-10 production and suppression of arthritis we determined the mRNA levels of the suppressors of cytokine signalling 1 and 3 (SOCS1 and SOCS3). The SOCS proteins are key negative regulators of cytokine responses and act via inhibition of the intracellular JAK/STAT signalling pathways [14], and IL-10 has previously been shown to induce these adaptor proteins [15]. We found elevated mRNA levels of SOCS1 and the same tendency (p = 0.12) also for SOCS3 in peripheral lymph nodes in LNT-IL-10 mice (Figure 2G). These data show that a local increase in IL-10 results in an increase in SOCS expression which correlates with suppression of arthritis development.

### LNT-IL-10 Influences Serum Protein Levels of Cytokines and Anti-CII Antibodies

**Figure 3:**
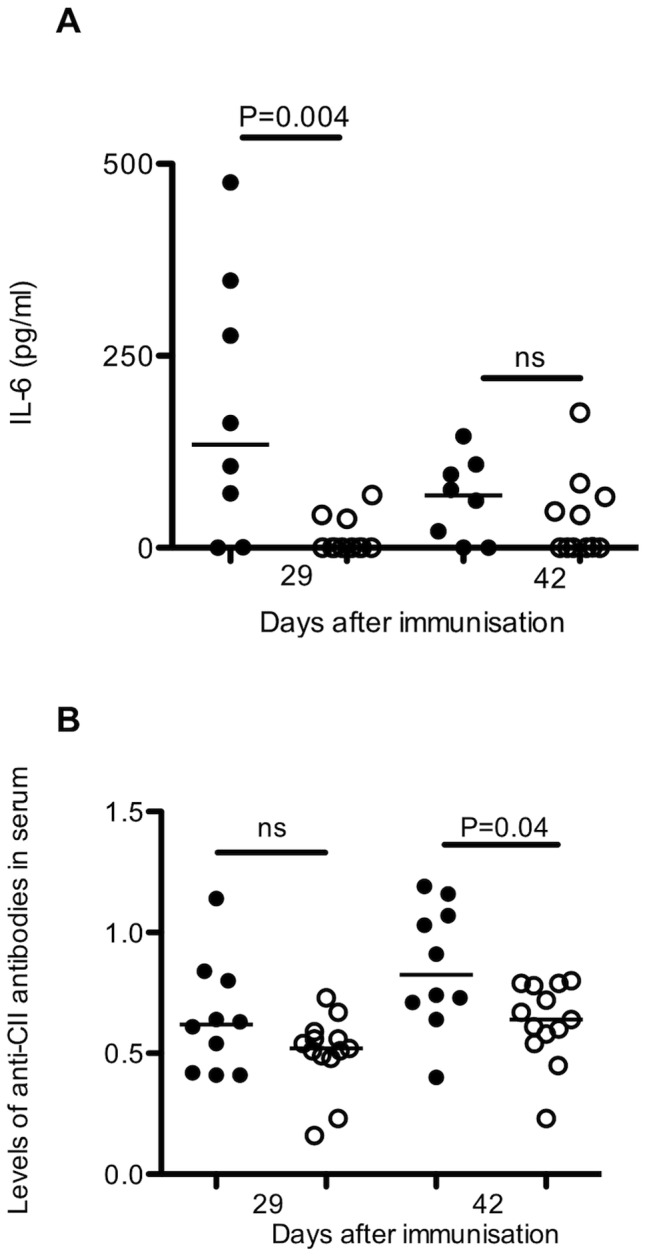
Levels of IL-6 and anti-CII antibodies (**A**) Serum protein levels of IL-6 (**B**) and serum levels of anti-CII IgG were analysed at days 29 and 42 after CII immunisation. Analysed by Mann-Whitney U-test. Closed circles represents LNT-GFP and open circles LNT-IL-10 mice.

The effect by IL-10 may be direct or indirect and we were, therefore, interested in potential effects on other cytokines. Indeed, we found a significant decrease in serum levels of IL-6 in LNT-IL-10 mice at day 29 after CII immunisation (Figure 3A). At day 42, although the levels were still very low in LNT-IL-10 mice, the levels of IL-6 in control mice had declined and the difference between the groups were no longer significant. Serum levels of a number of additional cytokines (IL-1α, IL-2, IL-4, IL-5, IL-10, IL-13, IL-17A, IL-21, IL-27, IFN-γ) were measured without any significant differences between the groups (data not shown). Previous work have shown that IL-6 promotes the development of arthritis as it together with TGF-β induces Th17 cells and stimulates B cells to increased production of IgG and IgA antibodies [16]. As may be expected, based on its effect on antibody producing cells, the serum levels of anti-CII specific IgG antibodies were decreased in LNT-IL-10 mice compared with LNT-GFP controls at days 42 and 49 (Figure 3B). These data demonstrate that an increase in local IL-10 expression in lymph nodes, but not in spleen, in LNT-IL-10 animals is accompanied by reduced serum levels of IL-6 and anti-CII antibodies. These findings indicate that local IL-10 production in the draining lymph nodes of arthritic joints acts via induction of an anti-inflammatory response that influences systemic cytokine levels and autoantibody production by B cells.

### LNT-IL-10 Influences Sizes of Cell Populations in Both Lymph Nodes and Spleen

**Figure 4:**
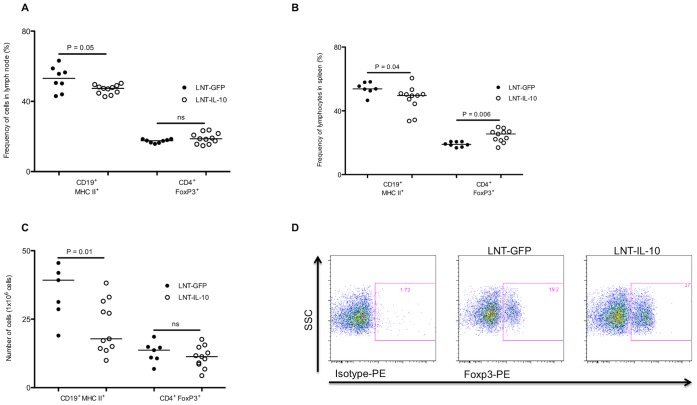
T and B cell populations in lymph nodes and spleen after CII immunisation. (**A**) Percentages of CD19^+^MHCII^+^ cells and CD4^+^FoxP3^+^ cells in lymph node, (**B**) and in spleen (**C**) Absolute numbers of CD19^+^MHCII^+^ cells and CD4^+^FoxP3^+^ cells in spleen. (**D**) Typical gating for isotype control and Foxp3 antibody in CD4^+^T cells from a LNT-GFP and a LNT-IL-10 mouse. All data were analysed by MannWhitney U-test. Closed circles represents LNT-GFP and open circles LNT-IL-10 mice.

IL-10 and IL-6 are potent inhibitors as well as activators of cell proliferation and differentiation. Because our data showed alterations in the levels of these cytokines between the groups we sought to determine whether this was associated with any differences in the proportions of B and T cells. Late during the course of arthritis (day 42) the proportion of CD19^+^ MHCII^+^ B cells was decreased in lymph nodes in LNT-IL-10 mice whereas the proportion of CD4^+^ Foxp3^+^ regulatory T cells was not affected (Figure 4A and D). At this late time point CIA has become a systemic disease and cell populations in spleen were also determined. In LNT-IL-10 mice the proportion of CD19^+^ MHCII^+^ B cells was decreased, and that of CD4^+^ Foxp3^+^ regulatory T cells increased (Figure 4B and D). However, in actual cell numbers this corresponded to an absolute decrease in B cells, but similar numbers of CD4^+^ Foxp3^+^ regulatory T cells as in controls (Figure 4C). Thus, the main effect of LNT-IL-10 appears to be on the B cell compartment.

## Discussion

Our report shows that increased local, but not systemic, levels of IL-10 conferred by disease-driven gene therapy delays the progression of CIA in mice. A precise and restricted increase in IL-10, produced by B cells and other APCs, ameliorates the course and severity of arthritis. Based on our data, a possible scenario would be that the increase in IL-10 upregulates SOCS1 resulting in a decrease in serum levels of IL-6. This in turn results in a decrease in both frequency and number of B cells and anti-CII antibody levels, accompanied by reduced severity of arthritis.

IL-10 is a potent pleiotropic cytokine that is produced e.g. by monocytes, macrophages, T and B cells. This cytokine has the capacity to inhibit synthesis of pro-inflammatory cytokines such as IL-2, IFN-γ, TNF-α and importantly IL-6 [4]. It has earlier been shown that systemically increased IL-10 levels suppresses the frequency and severity of CIA [17], [18], [19], [20], [21]. The inflammation-dependent IL-1/IL-6 promoter has low basal activity, which significantly increases during acute inflammatory conditions [13]. We found that this promoter, driving the IL-10 gene expression, does not induce increased systemic (serum) levels of IL-10 during the course of arthritis *in vivo,* but a locally increased IL-10 expression in lymph nodes; particularly in B cells and other APCs. Whether the B cells in the LNT-IL-10 mice are IL-10- producing regulatory B cells [17] is currently unknown, although it is possible as such cells have been found to reduce the severity of arthritis [18], [22]. Our data are supported by those of others [12], where it was recently found that a local and inflammation-dependent increase in IL-10 produced by endothelial cells results in suppressed development of zymosan induced arthritis in mice.

Interleukin-6 has been found to contribute to the development of synovitis as well as cartilage and bone destruction in autoimmune arthritis (reviewed in [23]). As expected, IL-6 was almost absent in the LNT-IL-10 mice but not in the arthritic control group. IL-6 is regulated by a multitude of mechanisms including SOCS1 and 3 e.g. SOCS1 down regulates its expression [24]. The SOCS adaptor proteins are in turn induced by IL-10 [15]. In fact, increased expression of both SOCS1 and 3 have been shown to decrease the severity of arthritis [15], [25], [26]. In our system, increased production of IL-10 in lymph nodes coincides with elevated mRNA levels of SOCS1 and decreased levels of IL-6. It is also known that IL-6 in the presence of TGF-β drives ROR-γt expression in naïve T cells to Th17 cells, while the absence of IL-6 induces FoxP3 expression and expansion of T regulatory cells [27], [28], [29]. At the studied time points, no differences in the number of T regulatory cells or serum levels of IL-17 could be detected, suggesting that this mechanism is less likely.

The frequency of B cells is decreased both locally in lymph nodes and systemically in spleen of LNT-IL-10 mice compared with controls. This effect might be attributed mainly to decreased IL-6 levels as the cytokine originally was identified as a B-cell differentiation factor and plays an important role in the development of antibody-producing plasma cells [30]. Beside the fact that fewer B cells can lead to lower levels of anti-CII IgG antibodies (which also could be due to a less inflammatory status), the beneficial effects of a reduced B cell population is well described in the outcome of human RA by the use of B cell depleting anti-CD20 antibodies [31].

Our study suggests that inflammation-dependent IL-10 production causing locally increased levels of IL-10, increased SOCS1 mRNA and a decrease in systemic IL-6 levels ameliorate the outcome of CIA in mice. However, the concept needs to be tested in human RA, as the role of IL-10 in RA patients is far from clarified: RA patients have significantly elevated levels of IL-10 in synovial fluid [32] while the expression of IL-10 receptors are reduced in synovial tissue [33] compared with osteoarthritic controls, and treatment with systemic recombinant IL-10 in human RA patients has so far not shown any convincing results [34]. Although these findings appear disappointing they do not contradict our data. Rather, they suggest that the anti-arthritogenic effect might be dependent on a requirement for localised rather than systemic IL-10 treatment.

**Taken together**, our study demonstrates that disease-regulated therapy mediated by IL-10 has a beneficial outcome on arthritis development in CIA and provides a step towards disease-regulated therapies in human autoimmune arthritis.

## Materials and Methods

### Cloning of Inflammation Dependent GFP and IL-10 Lentiviral Vectors

To generate the inflammation dependent lentiviral vector encoding the green flourescent protein (GFP), pGL3B containing the hybrid promoter (IL-1/IL-6) was digested with *BglII*/*Hind1I1.* The 1.3 kilo base pair (kbp) fragment was subcloned into a modified pBluescript vector, to have additional cloning sites, *Pac1/Asc1*. By using *Pac1/Asc1* the promoter fragment was further subcloned into pHR’SIN-cPPT-SEW containing both GFP and woodchuck post-transcriptional regulatory element (WPRE). The vector was named LNT-GFP. To create the inflammation dependent lentiviral encoding IL-10, the eGFP gene was digested from the LNT-GFP by *Pme1/Sal1*. A pCI vector containing the IL-10 cDNA was digested with *Not1/Xho1* and replaced the eGFP gene, creating the LNT-IL-10. All restriction enzymes and ligases were obtained from New England Biolabs (NEB Ipswich, MA, USA).

### Production of Lentiviral Particles

Vesicular stomatitis virus-G (VSV-G) pseudotyped lentivirus was produced by transient transfection of 293FT cells with three plasmids: one of the self inactivating transfer vector plasmids (LNT-GFP and LNT-IL-10); the multi-deleted packaging plasmid pCMVΔR8.74; and the VSV-G envelope pMD.G2 using calcium phosphate co-precipitation. At 72 h post transfection, the medium was harvested and concentrated by ultracentrifugation at 90,000 g. The pellets were resuspended in PBS containing 2% FCS and stored at −80°C.

### Lentiviral Particle Titration

Viral titer was determined on NIH/3T3 (American Type Culture Collection, Manassas, VA, USA) mouse fibroblast cell line using real time-PCR directed towards the WPRE sequence. Vector copy numbers are normalised to titin gene copies. WPRE forward primer: 5′ GGC ACT GAC AAT TCC GTG GT 3′, WPRE reverse primer: 5′ AGG GAC GTA GCA GAA GGA CG 3′ and WPRE probe 5′ 6-FAM- ACG TCC TTT CCA TGG CTG CTC GC- TAMRA- 3′. Titin forward primer: 5′ AAA ACG AGC AGT GAC GTG AGC 3′, titin reverse: 5′ TTC AGT CAT GCT GCT AGC GC 3′ and titin probe: 5′-6 FAM- TGC ACG GAA GCG TCT CGT CTC AGT C- TAMRA- 3′. All primers were obtained from Sigma-Aldrich AB (St Louis, MO, USA) and probes from Applied Biosystems and the assay was run with Taqman® Universal PCR Mastermix (Applied Biosystems, California, USA) on 7500 Real Time PCR System (Applied Biosystems).

### Mice

Male DBA/1 mice were obtained from Taconic (Europe A/S, Ry, Denmark) and housed in a pathogen-free barrier facility (12-hr light/12-hr dark cycle) and fed rodent chow. All animal studies were approved by the local Animal Ethics Committee.

### Inflammation-dependent Production of IL-10 *in vitro*

To verify inflammation-dependent IL-10 production, bone marrow was harvested from femur and the hip bone from DBA/1 mice and HSCs were isolated with negative selection using EasySep® Mouse Hematopoietic Progenitor Cell Enrichment Kit (Stemcell Technologies, Manchester, UK). After isolation, HSCs were resuspended in StemSpan with 1% penicillin/streptomycin and the following cytokines (100 ng/ml mSCF, 100 ng/ml Flt-3L, 100 ng/ml IL-11, 20 ng/ml IL-3) and cultured in 12 well plates at a concentration of 1 × 10^6^ cells/ml. The cells were transduced with lentiviral constructs LNT-GFP and LNT-IL-10 at MOI ranging from 0 to 80. The next day the media was changed to a media promoting differentiation of haematopoetic cells to bone marrow derived macrophages containing DMEM supplemented with 10% FCS, 10% L929- conditioned media, 20 mM HEPES and 50 µM 2-mercaptoethanol. After 9 days of differentiation the cells were stimulated with 100 ng/ml LipoPolySaccharide (LPS) or media for 24 h. Supernatants were collected and analysed by mouse Duoset IL-10 ELISA (R&D Systems, Abingdon, UK) according to the manufacturers instructions.

### Bone Marrow Transplantation

To minimize risk for infections during transplantation, both donor and recipient mice were treated with the antibiotic (enrofloxacin) Baytril® one week prior to and two weeks after the transplantation. Haematopoetic stem cells were harvested, isolated from donor mice as described in the paragraph above and further transduced with lentiviral constructs LNT-GFP and LNT-IL-10 at MOI 75 and incubated at 37°C overnight. The next morning, cells were washed with PBS twice, counted and resuspended at a concentration of 2.4 × 10^5^ cells/200 µl. The recipient mice were irradiated with 8.5 Gy and intravenously reconstituted with transduced HSCs (2.4 × 10^5^). Mice were repopulated for 12 weeks before induction of arthritis.

### Assessment of in vivo Transgene Integration by PCR

To detect vector integration in bone marrow, spleen and synovium 18 weeks after transplantation of transduced HSCs, DNA was prepared using the QIAamp DNA mini kit (Qiagen, Solna, Sweden) according to the manufacturer’s instructions and the WPRE was amplified with primers and probes described above.

### Collagen Type II Induced Arthritis

Two independent experiments were performed and the data were pooled. Arthritis was induced 12 weeks after bone marrow transplantation by a subcutaneous (sc) injection of chicken CII **(**Sigma-Aldrich AB) (1 mg/ml) in complete freund’s adjuvant (Sigma-Aldrich AB) in a total volume of 100 µl. The mice were boosted sc with CII (1 mg/ml, 100 µg/mouse) in incomplete freund’s adjuvant (Sigma-Aldrich AB) at day 21 after CII immunisation. All mice were followed individually and checked daily. Clinical arthritis and severity was assessed by an evaluator blinded to the treatment groups. Finger/toe and ankle/wrist joints were inspected and arthritis was defined as visible erythema and or swelling. To evaluate the severity of arthritis, a clinical scoring (arthritic index) was carried out using a system where macroscopic inspection yielded a score of 0–3 points for each limb. We define our scoring system as follows: 0– no arthritis, 1– mild arthritis (mild swelling and a subtle erythema of the evaluated joint), 2– moderate arthritis (moderate swelling and a more pronounced erythema compared to score 1), 3– severe arthritis (profound swelling and erythema). The total score per animal and time point is calculated by adding up the scores from all four paws. The mice were bled at day 29. At day 42 blood, joints, spleen and lymph nodes were obtained. Histopathologic examination of the joints was performed after routine fixation, decalcification, and paraffin embedding. Tissue sections from fore and hind paws were cut and stained with hematoxylin–eosin. All the slides were coded and evaluated by two blinded observers. The specimens were evaluated with regard to synovial hypertrophy, pannus formation, and cartilage/subchondral bone destruction. The degree of synovitis and destruction in every joint concerning finger/toes, wrists/ankles, elbows, and knees was assigned a score from 0 to 3. Occasionally one paw was missing in the histological sections, or embedded in such a way that it was impossible to evaluate the degree of synovitis and bone/cartilage destruction. Therefore, the total score per mouse was divided by the number of joints evaluated.

### Determination of mRNA Levels of IL-10 in Lymph Nodes

RNA was isolated from lymph nodes using RNEasy mini kit (Qiagen). The RNA quality was analysed using a Experion Bioanalyzer on a Experion RNA StdSens chip (Bio-Rad laboratories Inc., USA) prior to cDNA synthesis with High Capacity cDNA Reverse Transcription kit (Applied Biosystems). The expression of IL-10 gene was analysed using primers IL10 FW 5′-CATTTGAATTCCCTGGGTGAGA and RV 5′- TGCTCCACTGCCTTGCTCTT. The gene expression was normalised to β-actin analysed with primers FW 5′-CTGACAGGATGCAGAAGGAGATTACT and RV 5′-GCCACCGATCCACACAGAGT. The reactions were amplified using Power SYBR green PCR Master Mix (Applied Biosystems) and analysed on a Viia7 system (Applied Biosystems).

### Fluorescence Activated Cell Sorting (FACS) Analyses and Cell Counting

To detect intracellular cytokine expression in different cell populations, a single cell preparation of lymph node and spleen was performed. The total number of cells in spleen was counted (Nucleocounter, ChemoMetec AS, Denmark). For FACS stainings, 1×10^6^ from lymph nodes or spleen were placed in 96-well plates and pelleted (3 min, 300 g, 4°C). To avoid nonspecific binding via Fc-receptor interactions, cells were incubated with Fc-block (2.4G2, BD Biosciences, San Jose, CA, USA) for 10 min at room temperature. Antibodies used were anti-CD19 (clone ID3), anti-CD4 (clone RM4-5), anti-IL-10 (clone JES5-16E3) and anti-IFN-γ (clone XMG1.2) purchased from BD Biosciences, anti I-A/I-E (clone M5/114.15.2 purchased from BioLegend, San Diego, CA, USA) and FoxP3 (clone FJK-16s), and IL-17 (clone eBio17B7), purchased from eBioscience (San José, CA, USA). All surface marker antibodies were diluted in FACS-buffer (PBS containing, 1% FCS and 0.5 mM EDTA). For intracellular staining with anti-IL-10 or isotype controls the cells were permeabilised using the FoxP3/Transcription Factor Staining Buffer set from eBiosciences and antibodies diluted in 1×PERM buffer included in the kit. The antibodies were directly conjugated with fluorescein isothiocyanate (FITC), phycoerythin (PE), allophycocyanin (APC), V450 and APC-H7. Cells were stained as previously described and gating of cells was performed using fluorochrome minus one settings [35] and detected by FACSCanto II™ (BD Biosciences). Gating strategy for expression of IL-10 was included in Supplemental Figure 2. Analysis with respect to the number of cells and mean flourescence intensity (geometric mean) were performed using FlowJo Software, Tree Star Inc. (Ashland, OR, USA).

### Determination of SOCS Expression in Lymph Nodes

RNA was isolated from lymph nodes using RNEasy mini kit (QIAGEN). The RNA quality was analysed using a Experion Bioanalyzer on a Experion RNA StdSens chip (BioRad) prior to cDNA synthesis with High Capacity cDNA Reverse Transcription kit (Applied Biosystems). The gene expression of SOCS3 was analysed using primers FW 5′-CTGGTACTGAGCCGACCTCTCT-3′ and RV 5′-CCGTTGACAGTCTTCCGACAA-3′, the expression of SOCS1 was analysed using primers SOCS1 FW 5′-AAGGAACTCAGGTAGTCACGGAGTA-3′ RV 5′-CCGTGGGTCGCGAGAAC-3′. The gene expression was normalised to β-actin analysed with primers FW 5′-CTGACAGGATGCAGAAGGAGATTACT and RV 5′-GCCACCGATCCACACAGAGT. All reactions were amplified using Power SYBR green PCR Master Mix (Applied Biosystems) and analysed on a Viia7 system (Applied Biosystems).

### Determination of Cytokine Production

Blood was centrifuged at 7000 *g* for 10 min. Serum was collected and stored at −20°C for further analysis. Previously prepared spleen cell culture were stimulated for 72 hours with denatured chicken CII 50 µg/ml, supernatants collected and kept in −20°C until further analysed. Two independent experiments were performed. In the first experiment serum protein levels of IL-10 were measured in serum by Duoset ELISA (R&D systems) according to the manufacturer’s recommendations and detected on Spectra Max 340PC (Molecular Devices). In the second experiment serum levels of Th1, Th2, Th17 and Th22 specific cytokines (IL-1α, IL-2, IL-4, IL-5, IL-6, IL-10, IL-13, IL-17A, IL-21, IL-22, IL-27 and IFN-γ) were measured using FlowCytomix Multiple Analyte Detection Mouse Th1/Th2/Th17/Th22 13plex Kit (eBioscience). The assay was run on FACSCanto II™ (BD Biosciences). Analyses were performed using FlowJo Software, Tree Star Inc. (Ashland, OR, USA).

### Determination of the Anti-CII-specific IgG Antibodies

For quantification of anti-CII antibodies in serum, 96-well plates (Nunc, Roskilde, Denmark) were coated overnight at 4°C with 10 mg/ml of native chicken CII (Sigma-Aldrich AB). The samples were serially diluted (1∶250, 1∶750, 1∶2250, 1∶6750) in 0.5% bovine serum albumin (BSA) (Sigma-Aldrich AB) in PBS. Biotinylated F(ab•)2 fragments of goat anti-mouse IgG (Jackson Immuno Research Laboratories, Suffolk, England) were used as secondary antibody. Development was performed using horseradish peroxidase 0.5 µg/ml and 2.5 mg of the enzyme substrate 2,2-azino-bis-(3-ethylbenzothiazoline sulfonic acid) (Sigma-Aldrich AB) per ml in citrate buffer (pH 4.2), containing 0.0075% H_2_O_2_. The absorbance was measured at 405 nm on Spectra Max 340PC (Molecular Devices, Sunnyvale, CA, USA).

### Statistical Analysis

The levels of IL-10 in supernatants after treatment with LNT-GFP or LNT-IL-10 before and after LPS stimulation were compared using Two-way ANOVA (GraphPad Prism, GraphPad software, San Diego, CA, USA). All other statistical analysis between independent groups were calculated using the nonparametric Mann-Whitney U-test (GraphPad Prism) as described in the figure legends. A *P-*value ≤0.05 was regarded as being statistically significant.

## Supporting Information

Figure S1 **Integration of lentiviral vector and IL-10 production** ***in vitro*** **. (A)** The protein level of IL-10 in supernatants 9 days after in vitro transduction of HSCs with LNT-GFP or LNT-IL-10 at MOI 0, 40 or 80 and with or without LPS stimulation. (**B)** Integration of lentiviral vectors in bone marrow, spleen and synovial cells. The number of lentiviral particles LNT-GFP or LNT-IL-10 are expressed per 100 bone marrow cells, splenocytes or synovial cells. Data in figure 1A were analysed by Two-way ANOVA and data in figure 1B were analysed by Mann-Whitney U-test. Closed circles and black bars represents LNT-GFP and open circles and white bars LNT-IL-10 mice.
(TIFF)

Figure S2 **Gating strategy for detecting IL-10 expression in CD19^+^MHCII^+^ B cells using flow cytometry.**
(TIFF)
