# Supplementary figures and images for: Disease-Dependent Local IL-10 Production Ameliorates Collagen Induced Arthritis in Mice
Source: PLoS One. 2012 Nov 16;7(11):e49731. doi: 10.1371/journal.pone.0049731 (PMC3500327; doi:10.1371/journal.pone.0049731)

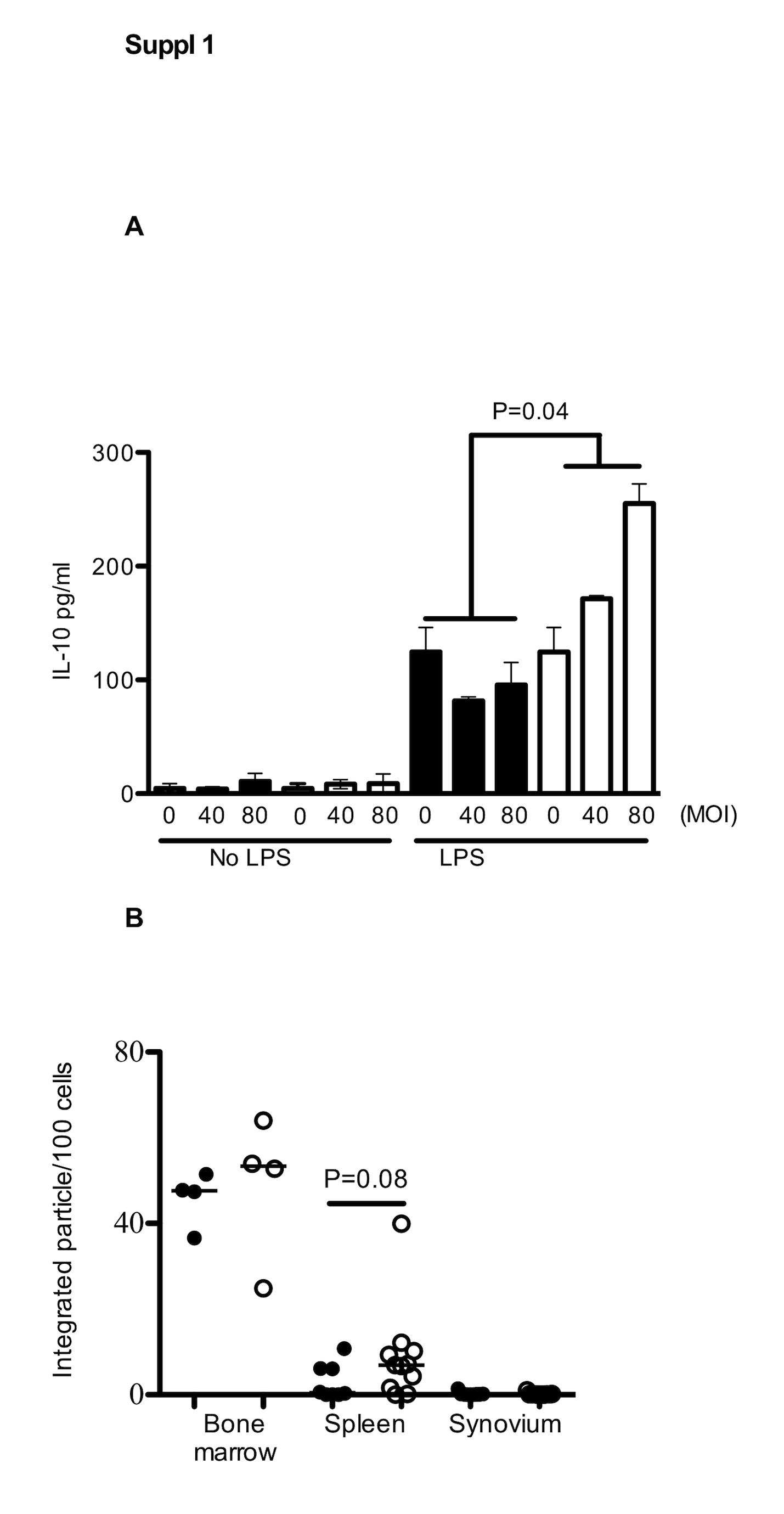

Supplement: Figure S1 — Integration of lentiviral vector and IL-10 production in vitro . (A) The protein level of IL-10 in supernatants 9 days after in vitro transduction of HSCs with LNT-GFP or LNT-IL-10 at MOI 0, 40 or 80 and with or without LPS stimulation. (B) Integration of lentiviral vectors in bone marrow, spleen and synovial cells. The number of lentiviral particles LNT-GFP or LNT-IL-10 are expressed per 100 bone marrow cells, splenocytes or synovial cells. Data in figure 1A were analysed by Two-way ANOVA and data in figure 1B were analysed by Mann-Whitney U-test. Closed circles and black bars represents LNT-GFP and open circles and white bars LNT-IL-10 mice. (TIFF) [file pone.0049731.s001.tiff]

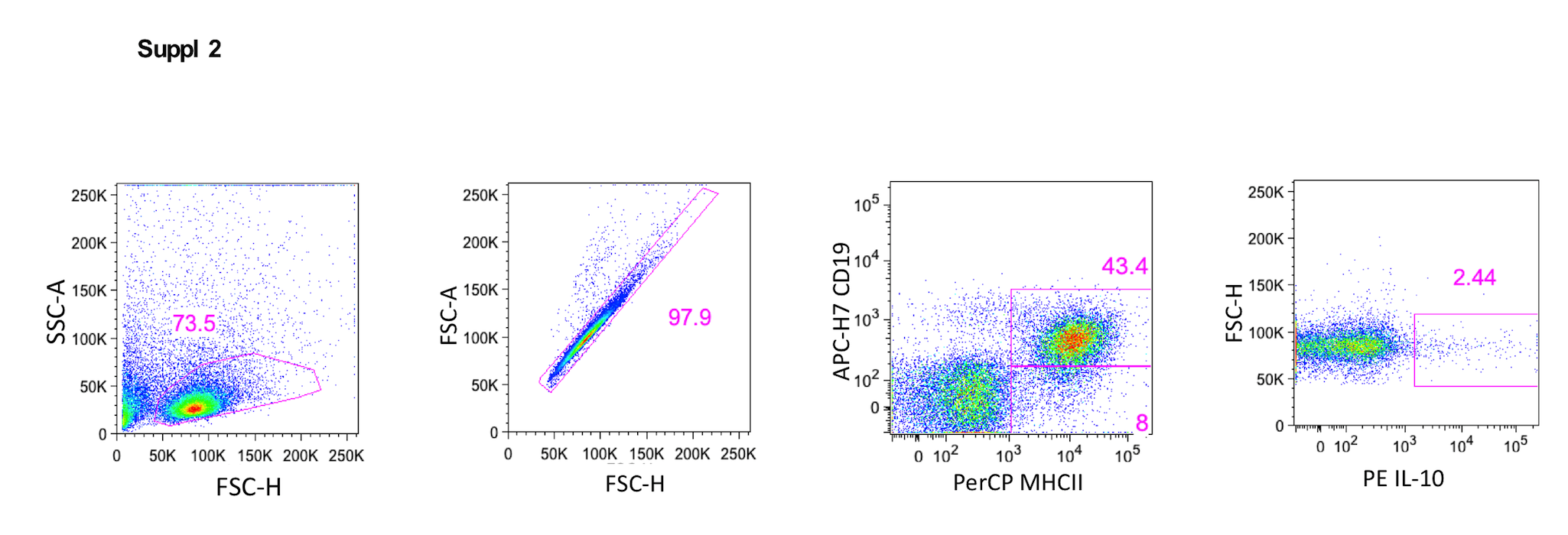

Supplement: Figure S2 — Gating strategy for detecting IL-10 expression in CD19+MHCII+ B cells using flow cytometry. (TIFF) [file pone.0049731.s002.tiff]
